# High-energy X-ray focusing and applications to pair distribution function investigation of Pt and Au nanoparticles at high pressures

**DOI:** 10.1038/srep21434

**Published:** 2016-02-23

**Authors:** Xinguo Hong, Lars Ehm, Zhong Zhong, Sanjit Ghose, Thomas S. Duffy, Donald J. Weidner

**Affiliations:** 1 Mineral Physics Institute, Stony Brook University, Stony Brook, NY 11794, USA; 2 National Synchrotron Light Source II, Brookhaven National Laboratory, Upton, NY 11973, USA; 3 Department of Geosciences, Princeton University, Princeton, NJ 08544, USA

## Abstract

We report development of micro-focusing optics for high-energy x-rays by combining a sagittally bent Laue crystal monchromator with Kirkpatrick-Baez (K–B) X-ray focusing mirrors. The optical system is able to provide a clean, high-flux X-ray beam suitable for pair distribution function (PDF) measurements at high pressure using a diamond anvil cell (DAC). A focused beam of moderate size (10–15 μm) has been achieved at energies of 66 and 81 keV. PDF data for nanocrystalline platinum (n-Pt) were collected at 12.5 GPa with a single 5 s X-ray exposure, showing that the *in-situ* compression, decompression, and relaxation behavior of samples in the DAC can be investigated with this technique. PDFs of n-Pt and nano Au (n-Au) under quasi-hydrostatic loading to as high as 71 GPa indicate the existence of substantial reduction of grain or domain size for Pt and Au nanoparticles at pressures below 10 GPa. The coupling of sagittally bent Laue crystals with K–B mirrors provides a useful means to focus high-energy synchrotron X-rays from a bending magnet or wiggler source.

X-ray microfocusing techniques for probing small sample volumes are important for a broad range of synchrotron radiation applications^1^,^2^. As the refractive index of most materials is close to unity in the X-ray range, focusing X-rays is challenging. However, there are today several types of optics for X-ray focusing utilizing different techniques including Kirkpatrick–Baez (K–B) mirrors^3^,^4^,^5^, capillary optics^6^, compound refractive lenses^7^, Fresnel zone plates^8^, and multilayer mirror optics^9^. These optics all have specific advantages and disadvantages depending on the experiment and employed X-ray energy. Most of these X-ray focusing devices are well-suited for moderate X-ray energies, but for higher X-ray energies (>30 keV), they lose efficiency because the refractive index decrement, δ = 1−n ~ 10^−6^, is small and scales as 1/E^2^, where E is the X-ray energy.

High-energy synchrotron radiation can be especially valuable for providing structural information covering short-to-medium range atomic order in crystals but this requires obtaining data covering a large Q range^10^,^11^, where Q = (4πsinθ)/λ is the scattering wave vector. The atomic pair distribution function (PDF) method using high-energy X-ray or neutron diffraction is a powerful tool for studying crystalline, disordered, and nanocrytalline materials^10^,^11^,^12^,^13^. Experimentally, the atomic pair distribution function, *G(r)*, can be obtained by Fourier transform, 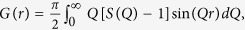 where *S(Q)* is the total scattering structure function. *S(Q)* to >20 Å^−1^ are desirable for high-pressure PDF, implying that short-wavelength, high energy X-rays or neutrons are required. However, reported high-pressure PDF (HP-PDF) data are sparse. Most studies to date use a small unfocused or slit-trimmed X-ray beam, and as a consequence are limited to relative low pressures. For example, HP-PDF measurements of GeO_2_ glass has been reported at pressures up to 15 GPa (115 keV/unfocused beam)^14^, 15.7 GPa (80 keV/trimmed beam)^15^, and 17.5 GPa (80.865 keV/trimmed beam)^12^, respectively. Furthermore, such studies typically require long time periods ( ~ hours) for data acquisition. For crystalline materials, current high-pressure PDF measurements are mainly limited to 10 GPa or less^16^,^17^. Development of optics for focusing high-energy X-rays (>60 keV) to achieve sufficient high flux for high-pressure PDF measurements is therefore desirable.

Available focusing optics for high-energy X-rays include refractive lenses^7^, K–B mirrors^18^,^19^ and bent Laue crystals^20^,^21^,^22^. Refractive optics are less effective at low energies, but are efficient for collimating/focusing high-energy X-rays (50–100 keV)^2^,^22^. However, the compound refractive lens has some limitations such as narrow angular acceptance, chromatic focusing, and long focal lengths^2^,^22^. Grazing-incidence, total-reflection K–B mirrors are widely used for microfocusing in the hard X-ray region^18^,^19^,^23^, due to the advantages of achromaticity, high efficiency, energy-tunability, and suitable working distances (~centimeters)^4^,^18^,^24^. The elliptical shape necessary for grazing incident X-ray optics can be achieved either by using a dynamical bender^4^,^18^,^24^ or differential deposition^3^. The main disadvantage of K–B optics is the low angular acceptance at high X-ray energies, leading to collection of only a relatively small portion of a wide incident beam by the mirrors. Despite this disadvantage, successful efforts to focus high energy X-rays by this method have been reported^18^,^19^.

A sagittally bent double-Laue crystal monochromator (DLM)^21^,^25^ has been implemented at high-energy X-ray beamlines such as the X17 beamline of the National Synchrotron Light Source (NSLS)^20^,^21^. Use of an anti-clastic bending method^20^,^21^ enables up to a ten-milliradian-wide incident fan source to be focused^21^,^25^. The X-ray flux density at the focal spot is a few hundred times larger than that of unfocused beams at energies of 20–50 keV^20^,^21^. However, the main limitation of the DLM geometry is that small spot sizes suitable for DAC experiments cannot be achieved.

For high-pressure PDF (HP-PDF) measurements utilizing DACs, the critical requirements include a high flux, small and clean X-ray beam, and high-energy radiation (> 60 keV). High flux is especially important for DAC experiments in which low diffraction intensity is normally the major experimental limitation. Focusing capability to~10 micrometers or less is the other key technical component, which allows the X-ray probe to be restricted to a small region of the sample and thus reduce the effects of pressure gradients as well as unwanted signal from the gasket, pressure standard, etc. Here we report the performance of a combined system involving sagittally bent Laue crystals and K–B mirrors to achieve high-energy X-ray micro-focusing and present applications to some selected high-pressure PDF measurements.

## Results and Discussion

### High-energy X-ray focusing optics

**Figure 1:**
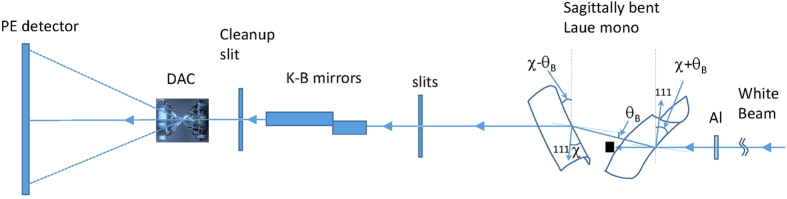
Schematic layout of high-energy X-ray focusing optics combining a sagittally bent Laue monochromator with K–B mirrors. The components from right to left (direction of X-ray propagation) are: aluminum (Al) filter, sagittally bent Laue crystals in anti-clastic bending geometry, slits, K–B mirrors, clean-up slits, DAC stage, and Perkin-Elmer (PE) detector.

The superconducting wiggler X-ray source at beamline X17of the NSLS, has a critical energy of 22 keV and provides high flux X-rays up to 100 keV with a horizontal fan of 18 mrad^26^. Figure 1 illustrates the schematic layout of the beamline and high-energy X-ray focusing optics, showing the arrangement of optical elements from the source to the detector.

Here, we provide a short summary of the DLM characteristics; a full description of the sagittally bent double-Laue optics can be found elsewhere^20^,^21^,^25^. The DLM consists of two bent Si (111) Laue crystals in anti-clastic layout (Fig. 1). 0.5-mm thick single-sided polished silicon (100) wafers were cut to a rectangular shape in the [011] direction. A four-bar bender was used to sagittally bend the planar crystal around the [011] axis^21^ (Fig. 1). The sagittal bending radius is changed by pushing a stainless steel leaf springs attached to the clamping blocks.

The white X-ray beam from the X17 superconducting wiggler source is monochromatized and initially focused by the DLM. This is followed by further focusing by a pair of trapezoidal K–B mirrors^4^. The incident white beam was set by slits to be about 5 mm (H) × 3 mm (V). An aluminum filter (2-mm thick) is employed to reduce the heat load on the monochromator. The DLM is located in the X17B1 hutch, 29 m from the source, and is located approximately 10 m upstream from the K–B mirrors. The DLM provides a slightly demagnified image of the source (around 1-mm horizontal size) at the location of the K–B mirrors in the downstream X17B3 station which is 39 m from the source. The DLM reduces the beam to approximately match the entrance aperture of the K–B mirrors. A slit is placed in front of the K–B mirrors to define an entrance aperture of 200 μm × 200 μm for the K–B mirrors.

The K–B system consists of two mirrors in tandem: a 100-mm long vertical mirror and a 200-mm long horizontal mirror. The mirrors are highly polished and composed of single-crystal silicon. A 100 Å layer of chromium is deposited onto the silicon substrates and serves as a binder for a 400-Å layer of Pt, which is deposited on top and acts as the X-ray reflecting surface. The mirror surfaces are dynamically bent to elliptical shapes by mechanical benders^4^,^24^.

Due to their high efficiencies and achromatic properties, K–B optics are often chosen as focusing optics for X-ray spot sizes down to 10 microns or less^23^. High reflectivity occurs at grazing incidence angles below the critical angle, θ_c_, which can be expressed as:


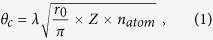


where λ is the X-ray wavelength, *r*_*0*_ is the Bohr atomic radius, and *Z* is the atomic number of the mirror coating layer. *n*_*atom*_ is the atomic concentration given by 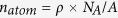, here *N*_*A*_ is the Avogadro’s number and *A* the atomic weight of the coating material. Higher critical energies result in smaller glancing incidence angles (Eq. 1). Therefore, high electron-density materials are preferable for the mirror surface to mitigate the reduction of critical angle, θ_c_, in the high-energy region. A platinum-coated mirror has a relatively large numerical aperture^18^,^19^. X-ray energies of 66 keV and 81 keV employed in this work correspond to incidence angles of 1.28 mrad and 1.04 mrad, respectively.

For an ideal elliptical focusing mirror, the focal length, f_KB_, is given by


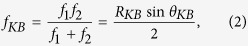


here *f*_*1*_ is the object distance, *f*_*2*_ is the image distance, *θ*_*KB*_ is the mirror angle and *R*_*KB*_ is the radius of curvature of the K–B mirror.

The DLM focal length is given by^20^


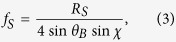


where *θ*_*B*_ is the Bragg angle of the monochromator, *R*_*s*_ is the sagittal bending radius and *χ* is the asymmetry angle of both Laue crystals (Fig. 1). It should be noted that the sagittal focal length is infinity when the crystal is not bent (*R*_*s*_ = ∞). The DLM focal length in the horizontal direction can be controlled by the applied bending^21^. Nevertheless, the sagittal radius *R*_*s*_ should not be too small (>20°), so as not to break the crystal^25^. The focal length, f_s_, is typically about 2–3 m^21^. In contrast, the K–B mirror has a much shorter focal length ( ~ 0.1 m)^4^.

The focal length, *f*, of the combined K–B mirror and DLM optics is therefore given by


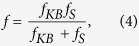


Since the value of DLM f_s_ is much larger than the focal length of K–B mirrors, i.e. f_s_ ≫ f_KB_, Eq. 4 can be simplified as


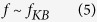


To block unwanted high-energy X-ray scattering, a clean-up pinhole (0.4-mm diameter pinhole in a 5-mm thick Pb disk) is placed between the K–B mirrors and the sample (Fig. 1). The combination of a DLM and KB mirrors can thus provide a focused microbeam at the sample with a convenient working distance while achieving intensity enhancement due to collection of a wide incident beam.

### Measurement of the focused beam profile

**Figure 2:**
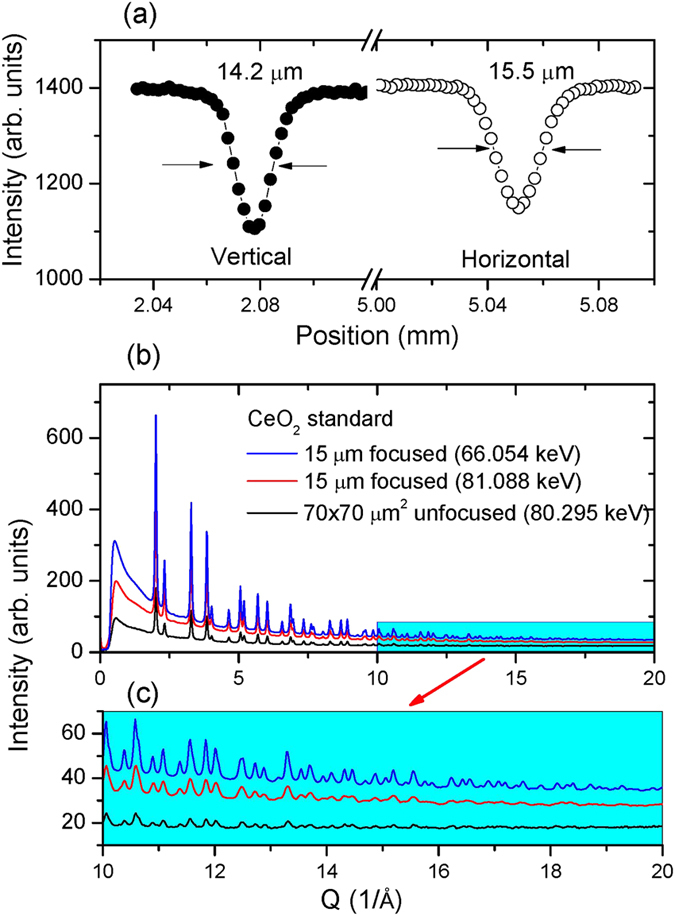
(**a**) Focused X-ray spot size in vertical and horizontal directions measured using a tungsten crosshair and a monochromatic X-ray beam at 81 keV; (**b**) Comparison of X-ray diffraction patterns of a CeO_2_ standard (loaded in ~30-μm thick gasket hole) measured using focused and unfocused beam, respectively; (**c**) Expanded view of high Q range (10–20 Å^−1^).

Figure 2a shows the beam profiles of a focused high-energy X-ray beam (81 keV) using the combined DLM and KB mirror system. The beam profile was measured by scanning across the beam in the horizontal and vertical directions using a relatively large tungsten crosshair (~15 μm size) for better absorption contrast with high energy X-rays. The best focused beam size achieved is ~15 μm full width at half maximum (FWHM). This experiment clearly indicates that the focused beam is convergent for the combined optics (Eq. 5).

Since the apparent size of 15 μm is the same as the crosshair diameter, the real beam size may be estimated from the base of the scanning profile (Fig. 2). Suppose the beam intensity is a Gaussian distribution, 
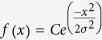
, where C is a constant and σ the standard deviation. The full width (FWHM) is 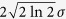, i.e. 2.355σ. By measuring the width of the intensity profile at *x* = *3σ* (inverse scanning profile, Fig. 2) and subtracting the crosshair diameter, the *σ* values of vertical and horizontal directions are 4.3 μm and 5.3 μm, respectively, which correspond a beam size of 10.1 μm (V) × 12.5 μm (H). Although these values are larger than the 3.3 μm size at 65 keV obtained by K–B mirrors with a smaller incident beam (70 μm)^18^, they are sufficient to conduct many DAC experiments up to the Mbar pressure range.

Figure 2b,c shows a comparison of X-ray diffraction patterns for a CeO_2_ standard by using focused and unfocused beams, respectively. The measurements were performed at ambient pressure with CeO_2_ loaded in a 250 μm hole of a ~30-μm thick Re gasket. It can be seen that the focusing optics leads to significant improvement in the overall diffraction intensity (Fig. 2b), especially for the data at high Q range (10–20 Å^−1^) (Fig. 2c), which is the prerequisite for conducting reliable PDF analysis and avoiding truncation error associated with the Fourier transform.

### High-pressure PDF applications

At ambient pressure, the PDF data of n-Pt (50 nm) can be fit well using a simple FCC Pt structure model with a small fit residual, *R*_*w*_, of 0.06 [see Fig. S1]. The measured lattice parameters are 3.9230 (1) Å and 3.9225 (1) Å by PDF fit and Rietveld refinement, respectively. The Rietveld value of 3.9225 (1) Å is somewhat larger than the 3.9178 Å for 10.6 nm Pt^27^, but slightly smaller than the bulk Pt of 3.9231 Å (JCPDS 00-004-0802). This implies that there is no significant structural modification due to the nano-size effect in n-Pt (50 nm) compared to bulk Pt.

**Figure 3:**
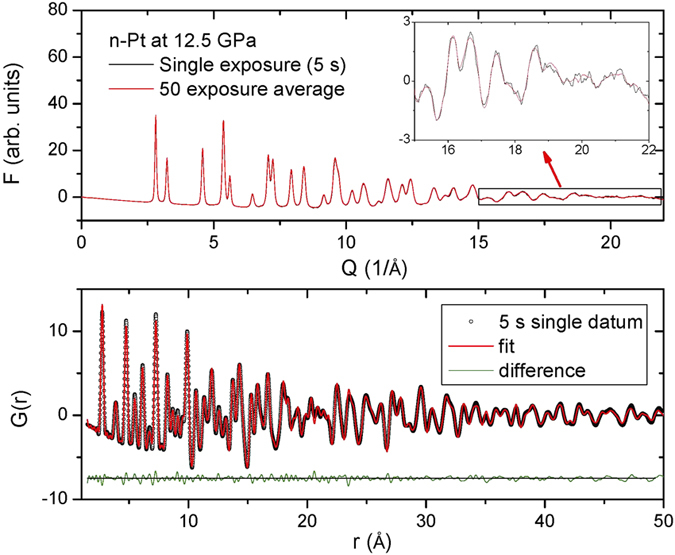
High-pressure pair distribution function (PDF) measurement of nano Pt (50 nm) at 12.5 GPa in M:E = 4:1 medium using a focused X-ray beam (66.054 keV). (Upper panel) Reduced total scattering function, *F(Q),* for a single 5-s exposure (black) and 50 averaged datasets (red). Inset shows an expanded view in the high-Q range. (Lower panel) Points represent PDF, *G(r)*, of a single 5 s exposure with *Q*_*max*_ = 21.5 Å^−1^ for the Fourier transform. Lines are the PDF fitting with a residual, *R*_*w*_, of 0.105. The difference curve shown at the bottom is offset for clarity.

Figure 3 shows the high-pressure pair distribution function (PDF) measurement of n-Pt (50 nm) at a pressure of 12.5 GPa in methanol/ethanol (4:1) medium using a focused X-ray beam (66.054 keV). In the high Q range, there is only a small difference in the total scattering function, *F(Q),* between a single 5-s scan and the averaged data accumulated over 50 scans (Inset, Fig. 3 upper panel). At this pressure, the PDF data can also be fit well using the simple FCC Pt structure model with a small fit residual (lower panel, Fig. 3). The high-quality of the fit suggests that the focused beam is of good quality, i.e. generally clean without harmonics or beam tail due to the combination of DLM and K–B optics. This may also be due to the effectiveness of the cleanup slit (Fig. 1). The measured lattice parameters are 3.88398 (26) Å and 3.88450 (26) Å with similar small fit residual, *R*_*w*_, of 0.105 and 0.102 for the single and averaged datasets, respectively. The high quality of our PDF data (5 s exposure) demonstrates that our system effectively combines the high flux of DLM and the small spot size of the K–B mirrors.

**Figure 4:**
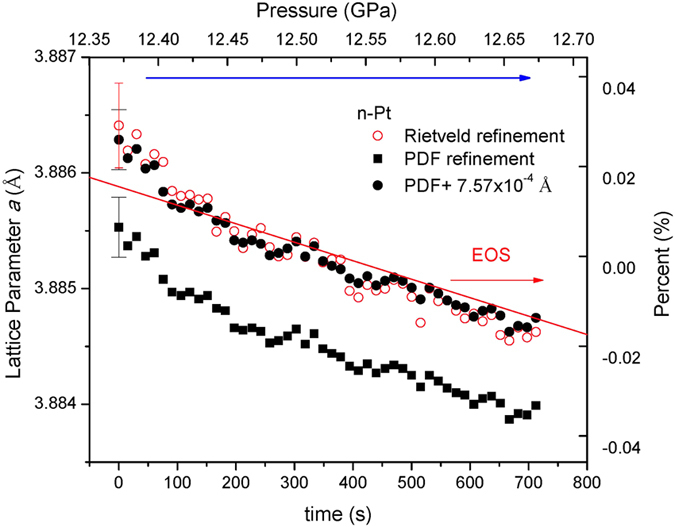
Real-time evolution of the lattice parameter of nano-crystalline Pt during a typical compression experiment. The systematic offset between PDF- and Rietveld-derived lattice parameter values is 7.57 × 10^−4^ Å. Averaged error bars are shown only at t = 0 s for clarity. EOS of bulk Pt (red line) is calculated using parameters from ref. 31.

The mechanical properties of nanoparticles are fundamental quantities in physics, engineering and geoscience. X-ray diffraction offers a unique way to probe these properties^28^,^29^. Figure 4 shows the evolution of the lattice parameter of n-Pt as a function of time during a typical compression experiment. The pressure values were measured using the ruby fluorescence technique^30^ before and after the PDF data collection at 12.5 GPa. For comparison, the equation of state (EOS) for bulk Pt (red line) is calculated using a Vinet equation with: the ambient-pressure volume, *V*_*0*_ = 60.38 Å^3^, isothermal bulk modulus, *K*_*0*_  = 277 GPa, and its pressure-derivative *K*_*0*_′ = 5.43, which are taken from ref. 31. It is notable that there is a small systematic deviation of 7.57 × 10^−4^ Å between PDF and Rietveld refinements for the same dataset, which may be associated with the different refinements in real and reciprocal space. Because almost all the EOS parameters have been based on Rietveld refinements so far, we then add the offset of 7.57 × 10^−4^ Å to the PDF data (solid circles, Figure 4), leading to a good agreement with the data of Rietveld refinements. To get rid of the influence of lattice stiffness caused either by size^27^,^28^ or microstructure (nanocrystallinity)^13^, the EOS curve at 12.35–12.70 GPa is normalized to the average value of Rietveld refinement (right axis, Fig. 4) for a direct comparison (right axis, Fig. 4). It can be seen that the evolution of the lattice parameter is basically in agreement with the EOS of bulk Pt over such a narrow pressure range (0.35 GPa). In many DAC compression experiments, the pressures were measured only once just before the X-ray diffraction. This result (Fig. 4) indicates that it is necessary to take into account the effect of DAC relaxation/enhancement or determine the lattice parameter and pressure simultaneously for a precise EOS determination of a material. The combined focusing optics (Fig. 1) provides intense flux for rapid data acquisition for PDF/diffraction experiments, which should be applicable for the EOS determination in rapid compression experiments.

**Figure 5:**
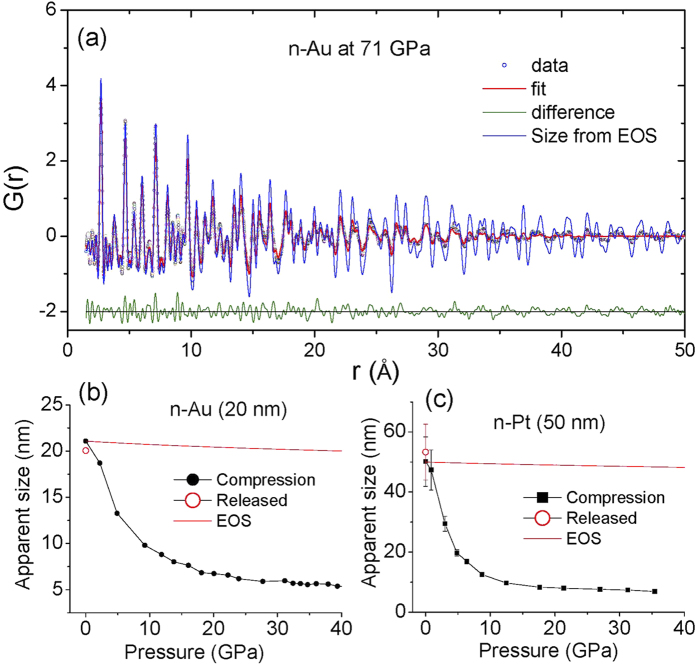
(**a**) PDF of nano-crystalline Au (20 nm) at 71 GPa using focused 81.400 keV X-rays. Points represent the *G(r)* up to *Q*_*max*_ = 20 Å^−1^ for the Fourier transform from an average of 200 individual measurements (5 s each). Lines are the PDF fitting with a residual, *R*_*w*_, of 0.244. The blue line shows the calculated PDF assuming the size of nano Au at 71 GPa is reduced from the ambient pressure value (20 nm) solely by the effect of compression as determined by the equation of state. (**b**) Evolution of the nano-domain size of n-Au (20 nm at ambient) compared to the effect of compression alone as given by the Au EOS^31^ (red line). The error bar is smaller than the symbol size. (**c**) Evolution of the nano-domain size of n-Pt (50 nm at ambient) as a function of pressure. Red line shows the reduction in nano-particle size due to compression using the EOS for Pt^31^. The open circles in (**b,c**) indicate data obtained on pressure release.

Figure 5a shows the HP-PDF measurement of n-Au (20 nm) at 71 GPa in a quasi-hydrostatic argon pressure-transmitting medium. The PDF fitting shows a relatively large fit residual, *R*_*w*_, of 0.244, implying that the structure of nano Au at 71 GPa deviates from the simple FCC structure. At 71 GPa, the volume of n-Au is 55.5 Å^3^, which is larger than the bulk one (54.2 Å^3^) calculated from ref. 31, but smaller than the EOS value of Gu *et al.*^28^ (56.5 Å^3^), suggesting a smaller sized-related stiffness as reported recently^13^. The blue line (Fig. 5) shows the calculated PDF based on the initial size of n-Au reduced by the effects of compression as determine from the EOS. It is clear that there is obvious pressure-induced extra damping in the *G(r)* function, which reflects a fundamental change of nanocrystallinity at high pressure^13^.

The size of a nanoparticle may play an important role in its optical, electronic, and mechanical properties due to quantum and structural confinement effects^28^,^29^,^32^,^33^,^34^,^35^. However, the nature of the internal structure of nanocrystals is not well understood^36^,^37^. In terms of PDF analysis^38^, Fig. 5b shows the evolution of the average grain or domain size of n-Au (20 nm at ambient) in an Ar pressure medium as a function of pressure, while Fig. 5c presents that of n-Pt (50 nm at ambient) loaded in a Ne medium, in comparison with calculated values based on their EOS^31^ (red lines). The striking observation is that the grain or domain size of both n-Pt and n-Au decreases substantially at pressures below 10 GPa at a rate which is much higher than that expected due to compression alone (top red lines), i.e. that the initial sample broke into much smaller grains. The breakdown in grain size is largely reversible upon pressure release (unfilled circles). This reversible behavior may reflect a pressure dependence of the large screw dislocations found recently in Pt nanopartiles^39^. Detailed analysis of the n-Pt microstructure and stiffness will be published in a separated paper while that of n-Au has been published recently^13^.

## Conclusion

We have shown that the coupling of a double-Laue crystal monochromator (DLM) with K–B mirror produces an efficient high energy X-ray focusing technique to produce a high-flux microfocused beam. The system can meet the stringent requirements of high-pressure pair distribution function measurement where the data quality at high Q range is the major concern. This focusing optics could be readily adapted to focus hard X-rays from a bending magnet or wiggler source. It should be applicable to a wide range of science including fundamental materials physics, geology, high-pressure research, and energy-materials research using high-energy focused X-rays. To address further scientific challenges, for example, ultrahigh pressure PDF, microstructure of advanced materials, and catalysis and materials processing, focusing the beam down to a size of 1–5 μm is a necessity in future work.

## Methods

Spherical Pt nano-particles (99.5%, <50 nm mean particle size) and Au nano-particles (99.9%, 10–20 nm mean particle size) were purchased from Sigma-Aldrich Co. and Nanostructured & Amorphous Materials, Inc., respectively.

High-pressure X-ray total scattering experiments were performed at beamline X17B3 of the National Synchrotron Light Source (NSLS), Brookhaven National Laboratory. The X-ray energy was calibrated using CeO_2_, Au and Si standards with typical energy deviations of 2 × 10^−4^ ΔE/E using a diffraction-based calibration method described elsewhere^40^. Once the X-ray energy is calibrated, the X-ray detector is set to a desired position for PDF/diffraction experiment. The sample-detector distance and detector orientation were then determined using the CeO_2_ standard with fixed X-ray energy.

Symmetric piston-cylinder diamond anvil cells (DAC) were used for pressure generation using diamonds with 300-μm diameter culets. The DAC had a downstream conical opening angle of~80 degrees providing an accessible range of *Q*-space up to 21.5 Å^−1^. Rhenium gaskets were pre-indented to a thickness of 50 μm, and a hole of 100-μm diameter was drilled to serve as a sample chamber. The n-Pt or n-Au powders were loaded as a loose powder to minimize particle contacts that are known to result in development of grain-to-grain micro-stresses. Methanol/ethanol (4:1 mixture by weight) and Ar were loaded as a pressure-transmitting medium for n-Pt and n-Au, respectively. Ruby chips were placed at the edge of sample chamber for pressure determination using the ruby fluorescence technique^30^.

The total X-ray scattering data were collected using a Perkin-Elmer (PE) flat panel detector (XRD 1261). This large-area detector is optimized for high-energy radiation and facilities data collection to high Q values but suffers from a relatively high dark current. In order to optimize counting statistics, alternating data and dark current series were collected, typically 50–200 datasets of each with an exposure time of 5 s and then averaged for dark current reduction to improve the statistical accuracy. The signal from a DAC with an empty gasket hole at ambient pressure, was used for background correction for measurements performed under high pressure.

The program Fit2D^41^ was used to integrate the raw intensity images. Any diffraction peaks from the diamond anvils or the pressure medium were masked prior to integration in FIT2D [see Figure S2]. Samples were compressed to a maximum pressure of 71 GPa for n-Au and 35 GPa for n-Pt. The integrated scattered intensity was corrected for background, Compton scattering, and absorption. The total scattering function, *S(Q),* and the pair distribution function, *G(r)*, were obtained using the program PDFgetX2^42^. The experimental PDFs were obtained by a Fourier transform of the total scattering structure function up to Q_max_ of 21.5 Å^−1^. The program PDFgui^43^ was used for PDF modeling and structural refinement using data in the range from 1–50 Å. For the same data sets, Rietveld refinements of X-ray diffraction spectra were performed using the program Jana2006^44^.

Due to the finite crystallite size and disorder inherent in nanoparticles, the *G(r)* function exhibits attenuation associated with particle size. The PDF of a spherical nanoparticle of size *D* can be expressed as^38^:


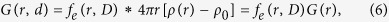



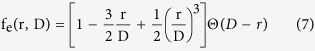


where 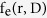 is the damping envelope function^45^ and Θ(x) is the Heaviside step function. For size determination, we first refine the PDF of the bulk CeO_2_ standard using PDFgui to determine the instrument parameters related to broadening, *Q*_*broad*_, and damping, *Q*_*damp*_. The particle diameter can be obtained by fitting the *G(r)* of n-Au, whose oscillations are attenuated by the envelope function (Eq. 7).

## Additional Information

**How to cite this article**: Hong, X. *et al.* High-energy X-ray focusing and applications to pair distribution function investigation of Pt and Au nanoparticles at high pressures. *Sci. Rep.* **6**, 21434; doi: 10.1038/srep21434 (2016).

## Supplementary Material

Supplementary Information
