# Supplementary material for: High-energy X-ray focusing and applications to pair distribution function investigation of Pt and Au nanoparticles at high pressures
Source: Sci Rep. 2016 Feb 23;6:21434. doi: 10.1038/srep21434 (PMC4763265; doi:10.1038/srep21434)
Supplement: Supplementary Information [file srep21434-s1.pdf]

Supporting Information

High-energy X-ray focusing and applications to pair distribution function investigation of Pt and Au nanoparticles at high pressures

Xinguo Hong[1,*], Lars Ehm[1,2], Zhong Zhong[2], Sanjit Ghose[2], Thomas S. Duffy[3,*] and Donald J. Weidner[1]

[1]Mineral Physics Institute, Stony Brook University, Stony Brook, NY 11794, USA

[2]National Synchrotron Light Source II, Brookhaven National Laboratory, Upton, NY 11973, USA

[3]Department of Geosciences, Princeton University, Princeton, NJ 08544, USA

Supplementary Figures

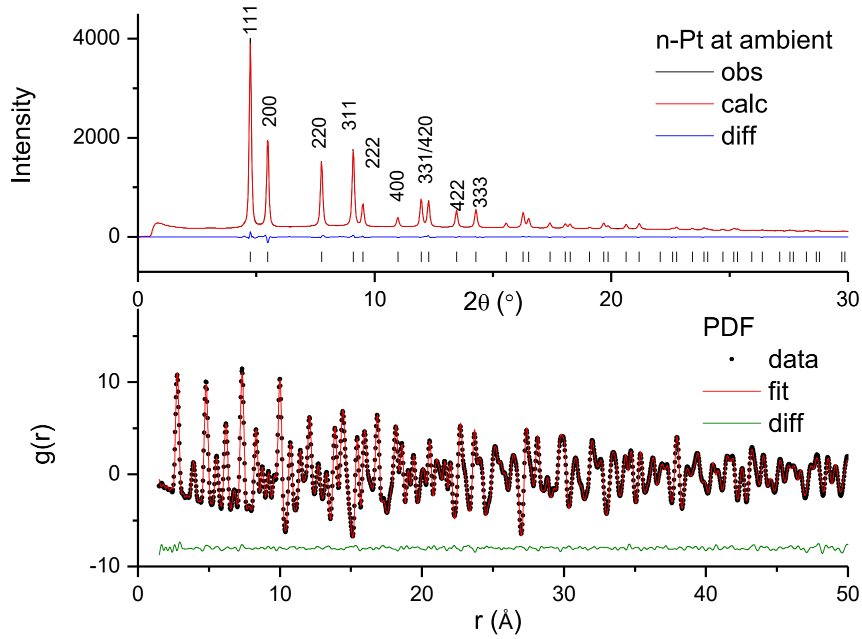

Figure S1.

(Upper panel) Rietveld refinements of the diffraction patterns of n-Pt (50nm) at ambient pressure using a simple FCC Pt structure model with a fit residual of R_{obs}: 2.32, ωR_{obs}: 3.02; and GoF_{obs}: 0.24. The observed, calculated and difference curves are represented. The tick marks indicate the calculated Bragg reflections. The lattice parameter refinements based on the simple FCC model shows a slight misfit in the intensity ratio of the 111 and 200 reflections.

(Lower panel) the corresponding high-pressure PDF fit using a simple FCC Pt structure model for the same diffraction data with a fit residual, R_w, of 0.06, in the range of 1.5-50 Å. The experimental g(r), calculated and difference curves are represented.

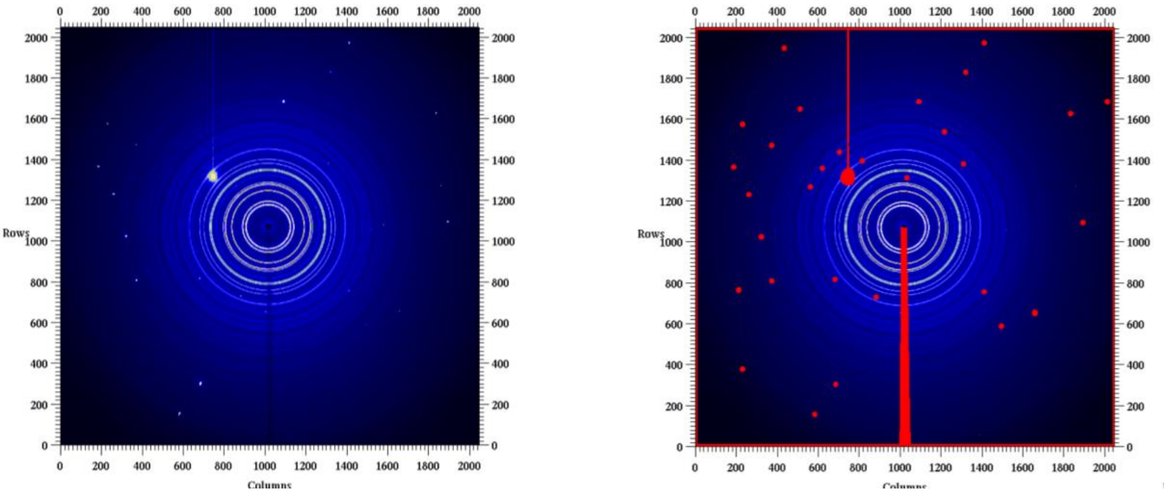

(a) (b)

Figure S2. X-ray diffraction image of nano Pt (50 nm) loaded in M:E=4:1 medium at 12.5 GPa using a focused X-ray beam (66.054 keV). (a) Raw image; (b) image with masked unwanted peaks for Fit2D integration.
